# Multidimensional Loneliness Among University Students: A Latent Profile Approach

**DOI:** 10.3390/ijerph23010050

**Published:** 2025-12-31

**Authors:** Aditya Banerjee, Neena Kohli, Sarabjeet Kaur Chawla, Vrrinda Kohli

**Affiliations:** 1 Department of Liberal Arts Humanities & Social Sciences, Manipal Academy of Higher Education, Manipal 576104, India; aditya.banerjee@manipal.edu; 2 Department of Psychology, University of Allahabad, Prayagraj 211002, India; 3 Independent Researcher, Prayagraj 211002, India; vrrindakohli03@gmail.com

**Keywords:** latent profile analysis, loneliness, depression, personality

## Abstract

Background: An increasing number of university students report feeling lonely, a negative experience arising from a mismatch between perceived and actual social relationships. Loneliness has been linked to poorer mental health. However, the relationship between qualitative (sources of loneliness) and quantitative (high or low) differences in loneliness and mental health is under researched. The aims of this research were to (a) identify profiles of loneliness among university students across three indicators of loneliness, namely, social, family, and romantic indicators, using latent profile analysis (LPA); (b) examine the differences among identified profiles based on dimensions of mental health indicators (depression, anxiety, and stress), social support, and life satisfaction; and (c) assess profile membership based on demographic variables (gender, social isolation, relationship status, and education characteristics) and the Big Five personality traits (extraversion, agreeableness, openness, conscientiousness, and neuroticism). Method: A cross-sectional survey was conducted on 912 university students from five cities in Uttar Pradesh, India. Participants completed questionnaires covering demographic details and validated measures assessing loneliness, depression, stress, anxiety, social support, life satisfaction, and the Big Five personality traits. Data were analyzed using the latent profile module in Jamovi and fit indices, namely, BIC, AIC, and BLRT, and entropy was used to select the best profile. Results: The latent profile analysis identified four profiles for university student loneliness, including Social and emotional lonely (31.4%), Moderate romantic lonely (23.8%), Moderate social lonely (8.2%), and Severe romantic lonely (36.6%). Moreover, the Social and emotional lonely profile scored the highest on depression, anxiety, and stress. The Moderate romantic lonely profile scored the highest on life satisfaction and social support. Being in a relationship decreased the likelihood of being categorized as Severe romantic lonely. In terms of personality, neuroticism was the strongest predictor of profile membership. This study is a step towards identifying at-risk lonely individuals with varying sources of loneliness. Identifying different profiles of lonely individuals will have direct implications for designing interventions that cater to a particular group rather than a one-size-fits-all approach.

## 1. Introduction

### 1.1. Loneliness as a Problem

Loneliness is a major problem affecting society in the 21st century [1,2]. The global risk report from the World Economic Forum (WEF) recognizes loneliness as a major risk that could have significant adverse impacts on several countries or industries over the next ten years [3]. The negative consequences of loneliness are comparable to those of smoking 15 cigarettes a day [4] and place a significant economic burden on society [5,6,7]. Although loneliness can be experienced throughout life, a high prevalence of loneliness among university students, ranging from 23.6% to 32.6%, has been reported across global north countries [8,9,10,11]. However, countries in the global south, such as India, are also experiencing loneliness-related problems, though there is a lack of empirical evidence on the state of loneliness, especially among university students in India [12,13,14].

There are conflicting viewpoints among researchers defining loneliness. The prominent perspective considers loneliness to be unidimensional, describing it as an experience that varies only in intensity [15]. However, the multidimensional perspective of loneliness states that it varies in terms of intensity and source among individuals. Weiss [16] argued that loneliness consisted of multiple dimensions and introduced the concept of two different subtypes of loneliness: emotional loneliness and social loneliness. Emotional loneliness occurs when an individual feels isolated from those around them and may not have an intimate connection with others as they might like [17]. In contrast, social loneliness typically refers to a lack of social networks that the individual can tap into to receive support [8]. Within this classification of loneliness, some have argued that the types of loneliness can be divided into even more subtypes; for example, that emotional loneliness can be broken down into romantic and family loneliness [18]. Research has shown that the components of multidimensional loneliness differ around emerging adulthood [19]. Emotional (romantic and family) loneliness increases around emerging adulthood, and social loneliness decreases [8,20].

### 1.2. Loneliness from a Person-Centric Approach

Loneliness is often examined using variable-centric approaches that assess average scores across a population. Such methods may obscure meaningful individual differences in how loneliness is experienced, for example, due to variations in levels of social, romantic and family loneliness. These variations cannot be captured adequately by single global loneliness score. To address this challenge, a parallel growing stream of research explores loneliness by identifying sub-populations with varying intensities or sources of loneliness. These ‘person-centric’ studies on loneliness are in their infancy compared to ‘variable-centric’ studies. However, the advantage of using a person-centric technique is identifying various sub-populations with unique characteristics [21]. In the context of loneliness research, individuals may be categorized into different profiles with unique patterns of loneliness across dimensions. For example, a study in Northern Taiwan reported three cluster profiles among young adults with varying degrees of emotional and social loneliness [22]. Similarly, Hsu [23] reported four clusters of lonely older adults in a large sample in Taipei. The research used cluster analysis to classify a sub-population of lonely individuals. The drawback of such techniques is that no statistical criteria exist to identify the number of clusters. On the other hand, more sophisticated person-centred techniques such as latent profile analysis (LPA) and latent class analysis (LCA) provide a probability-based statistical method for determining an optimal number of profiles. Different loneliness profiles have been identified using the LPA/LCA methods with a minimum of two profiles [24]. Similarly, some studies have also identified three [25,26], four [27,28,29,30,31,32], and five profiles [33,34].

The studies have primarily been conducted in developed countries such as the USA [28,29], China [26,33], and some European countries such as Belgium [30] and Sweden [34]. Most LPA/LCA studies have identified different profiles of lonely individuals based on differences in the intensity, frequency, and duration of loneliness. A study on university students during COVID-19 identified three different profiles: low loneliness, interpersonal sensitivity loneliness (moderate loneliness), and high loneliness [26]. Similarly, another study on young adults in the US reported four profiles: lonely and disconnected, moderately lonely and disconnected, ambivalent feelings, and connected and not lonely [27].

In terms of multi-dimensional studies on loneliness, a study among young adults in China identified three profiles: emotional loners, serious emotional loners, and severe emotional/social loners [25]. Similarly, another study on US adults reported four different profiles of loneliness with varying intensity and qualitative differences: low lonely, social lonely, emotional lonely, and social–emotional lonely profile [29]. Research has also shown that multi-dimensional loneliness profiles vary across different psychopathologies. A study by Chau et al. [33] identified five different profiles. Individuals with high loneliness across intimate, relational, and collective loneliness also scored highly across symptoms of depression, social anxiety, and paranoia. Similarly, a study also reported that profiles reporting multiple sources of loneliness have higher odds of developing depressive symptoms, suicidal tendencies, and psychological distress [25,29].

### 1.3. The Current Study

The study addresses the problem of multi-dimensional loneliness among university students using a person-centred technique, which focuses on individuals rather than the sample as the unit of analysis by identifying profiles of loneliness in university students. The study did not have a specific hypothesis about the characteristics of the profiles due to conflicting reports in the existing literature. However, it was anticipated that profiles would vary across family, romantic, and social loneliness dimensions. The study subsequently examines the validity of profiles by comparing the profiles across indicators of mental health and well-being. The profiles may differ in depression, anxiety, stress, social support, and life satisfaction. The study also examines the profile prediction and odds ratio between profiles and demographic and Big Five personality traits. The objectives of this study were (1) to examine whether different profiles of lonely university students exist among a representative sample of university students; (2) to examine the differences among identified profiles across dimensions of mental health indicators (depression, anxiety, and stress), social support, and life-satisfaction; and (3) to assess the profile membership of demographic variables (gender, social isolation, relationship status, and education characteristics) and Big Five personality traits (extraversion, agreeableness, openness, conscientiousness, and neuroticism).

## 2. Materials and Methods

### 2.1. Participants

A convenience sampling technique was employed to recruit participants. Forms were given to 1028 participants, of which data from 945 participants were received (91.9%). The sample size fulfilled the criteria of *n* > 500, which is a prerequisite for latent profile analysis [35]. The data was collected during the timeframe of two months (October–November 2022). During data analysis, 33 (3.5%) participants were dropped due to (1) missing gender (*n* = 12) and (2) missing values identified during the data cleaning process (*n* = 21).

The final sample consisted of 912 participants. The mean age of the participants was 21.46 (2.49), with 451 males (49.5%) and 461 females (50.5%). The sample was drawn from university students from five cities in Uttar Pradesh, India—Bhadohi (*n* = 98, 10.7%), Kanpur (*n* = 100, 11%), Lucknow (*n* = 95, 10.4%), Prayagraj (*n* = 510, 55.9%), and Varanasi (*n* = 109, 12%). Regarding relationship status, most were single (*n* = 723, 79.3%). The current living situations of the participants were living alone (150, 16.4%) and living with parents/roommates or extended family (*n* = 762, 83.6%). In total, 468 (51%) of the participants lived away from their homes for university.

### 2.2. Procedure

A call for participants was advertised for graduate and post-graduate students in universities in five cities in Uttar Pradesh. Participants who showed interest in the research were invited to participate. The participants were also informed that their confidentiality would be maintained throughout the coding process; no personal identifiers were used. Email ID and phone number were only collected for future reference. The interested participants were then handed informed consent forms. The form briefly detailed the study’s aim, purpose, risks, benefits, and a confidentiality statement. After giving their consent, the participants proceeded to participate in the study. The time taken to fill out the form varied from 15 to 20 min.

### 2.3. Measures

The questionnaire consisted of standardized measures and a section on the participant’s demographic information. Demographic details included age, gender, socio-economic status, area where they had grown up, family type, relationship status, social isolation, year at university, and stream of education.

#### 2.3.1. The Social and Emotional Loneliness Scale for Adults (SELSA)

Loneliness was assessed with the short version of the Social and Emotional Loneliness Scale for Adults [18,36]. The SELSA-S measures loneliness using fifteen statements across three sources of loneliness—romantic, family, and social loneliness—over the past year. The items are rated on a 7-point scale, ranging from 1—strongly disagree to 7—strongly agree. The scale is a reliable multi-dimensional measure of loneliness and has been used extensively with the university population [37,38]. The scale has been previously used with Indian university students [39]. The Cronbach’s alpha in the current study for family loneliness is 0.71, romantic loneliness 0.69, and social loneliness 0.70.

#### 2.3.2. Multi-Dimensional Scale of Perceived Social Support (MSPSS)

Perceived social support was assessed using the Multi-dimensional Scale of Perceived Social Support [40]. This scale consists of 12 items to measure social support from friends, family, and significant others. Each sub-scale consists of four items with response options ranging from 1 (very strongly disagree) to 7 (very strongly agree). Past studies on social support have also used this scale as a unidimensional measure based on the aggregate score of three support dimensions [41] with an internal consistency of 0.91. The current study also measures social support as an aggregate score of three dimensions. The Cronbach’s alpha coefficient for the present study is 0.89.

#### 2.3.3. Depression, Anxiety, and Stress Scale (DASS-21)

Depression, anxiety, and stress were measured using the DASS-21 [42]. The scale consists of 21 items measured on a 4-point Likert-type scale to measure depression, anxiety, and stress. Each subscale consists of seven items. The depression subscale assesses a lack of interest/involvement, devaluation of life, and hopelessness. The anxiety subscale assesses anxious affect, skeletal muscle effects, and autonomic arousal. The stress subscale assesses difficulty relaxing, nervous arousal, being easily agitated, and over-reactive behaviour. The Cronbach’s alpha coefficient in the current study for depression (α = 0.85), anxiety (α = 0.79), and stress (α = 0.83).

#### 2.3.4. Satisfaction with Life Scale (SWLS)

Life satisfaction was measured using the Satisfaction with Life Scale [43]. This scale provides an aggregate score of satisfaction with life, with participants responding to items on a 7-point Likert scale. The Satisfaction with Life Scale has been widely used in research and demonstrates good psychometric properties with college students [44]. A few studies have pointed out that the first three items of the scale are better indicators of present satisfaction with life, and the last two measure satisfaction with one’s past [45]. The Cronbach’s alpha coefficient in the current study for SWLS is 0.83.

#### 2.3.5. The Big Five Inventory 10 (BFI-10)

Personality was measured using the Big Five inventory 10 developed by Rammstedt and John [46]. It is a short version of the well-established Big Five Inventory [47]. The original scale consisted of 44 standard BFI items. BFI-10 measures personality using only two items per dimension—openness to experience, conscientiousness, extraversion, agreeableness, and neuroticism. All items are rated on a five-point Likert-type with response options ranging from fully agree to fully disagree. Past studies have shown that BFI-10 shows a low Cronbach’s alpha; for example, a study reported internal reliability ranging from 0.24 to 0.64 from a study on Romanian university students [48]. Similarly, Thalmayer et al. [49] reported an alpha range of 0.37 to 0.72, and Credé et al. [50] reported 0.37 to 0.65. In the present study, Cronbach’s alpha ranged from 0.35 to 0.68.

### 2.4. Data Analysis

#### 2.4.1. Determining Profiles of Multidimensional Loneliness

Latent profile analysis was conducted on Jamovi v. 2.2.5 using the snowRMM v.5.2.1’ module. The latent profile analysis runs on the TidyLPA package for R [51]. LPA was estimated using three SELSA-S subscales: family, romantic, and social loneliness. Four latent profile models were estimated based on different parameter constraints for means, variances, and covariances [51,52]. Specifically, the models were Model 1—equal variances, covariances fixed to zero; Model 2—varying variances, covariances fixed to zero; Model 3—equal variances and equal covariances; and Model 6—varying variances and covariances. Models 4 and 5 can only be fit using premium software such as MPlus. Since the scope of the study is exploratory, all models were explored, and the best-fit model was chosen based on recommendations by past LPA researchers [35,53]. The following indices were used to judge the optimal solution—models with lowest scores on the Bayesian Information Criterion (BIC) [54] and Akaike Information Criterion (AIC) [55] were judged to have the best fit scores. Latent profiles were generated until there was no further reduction in the information criterion. A significant Bootstrapped likelihood ratio test (BLRT) indicated improvement over previous models [56]. A high entropy score indicated pure model [57]. The class membership percentage of *n* > 5% was considered the minimum percentage of each profile [53].

#### 2.4.2. Differences Between Mental Health Outcomes

After determining the optimal number of latent profiles, differences between extracted latent profiles as fixed factors and depression, anxiety, stress, social support, and life satisfaction as dependent factors, the data was analyzed using one-way ANOVA or Welch’s ANOVA (W-test) with Games–Howell post hoc tests when the assumption of homogeneity of variance was breached.

#### 2.4.3. Profile Membership of Personality

Profile membership was determined using multinomial logistic regression to determine whether demographic variables (gender, living situation, relationship status, and education characteristics) and Big Five personality traits (openness to experience, conscientiousness, extraversion, agreeableness, and neuroticism) predicted the profile membership. The prediction of class membership was considered significant when *p* < 0.05. The researcher also checked whether any variable increased the odds of classifying a particular profile through the odds ratio of profile membership.

## 3. Results

Table 1 shows correlation among study variables. Table 2 describes the fit indices of the models generated. The entropy values were higher for two- and three-profile solutions (0.88 and 0.89). However, the Bayesian Information Criterion (BIC) and Akaike Information Criterion showed that four latent profiles of varying variance and covariance fixed to 0 (model 2) was the most parsimonious model with a moderate-to-high certainty of classification (entropy = 0.73). This profile combination showed the lowest BIC and AIC values compared to other models. Additionally, the significant BLRT *p*-value indicates an improvement in model estimation compared to other models.

Table 3 describes the detailed characteristics of the four latent profiles. Profile 1 (*n* = 286, 31.4%) was characterized by the highest score on family loneliness, a moderate score on social loneliness, and the highest score on social loneliness. This profile was named the social and emotional lonely group. The lowest family loneliness, moderate romantic loneliness, and the lowest social loneliness scores characterized the second profile (*n* = 217, 23.8%). Based on the elevated score in the romantic dimension, the profile was named the moderate romantic lonely group. The third profile (*n* = 75, 8.2%) was characterized by low family loneliness, the lowest romantic loneliness, and moderate social loneliness and was named the moderate social lonely group. The fourth profile (*n* = 334, 36.6%) scored low on family loneliness, had the highest romantic loneliness, and moderate social loneliness. Based on its high score on the romantic dimension, the profile was named the severe romantic lonely group.

In Table 4, Welch’s ANOVA revealed that there were significant differences among depression scores across profiles (3, 304.08) = 35.74, *p* < 0.001, η^2^ = 0.11. The post hoc Games–Howell test indicated significant differences in depression scores across profiles (all *p*-values < 0.05) except for profile 2 (moderate romantic lonely) vs. profile 3 (moderate social lonely). Similarly, there were significant differences among anxiety scores. Welch’s statistic (3, 297) = 17.1, *p* < 0.001, η^2^ = 0.05. Post hoc comparisons indicated significant differences in anxiety scores across profiles (all *p*-values < 0.05) except for profile 2 (moderate romantic lonely) vs. profile 3 (moderate social lonely). Welch’s statistic for stress was also significant with (3, 296) = 15.4, *p* < 0.001, and η^2^ = 0.05. The post hoc Games–Howell test indicated significant differences in stress scores across profiles except for profile 2 (moderate romantic lonely) vs. profile 3 (moderate social lonely) and profile 3 (moderate social lonely) vs. profile 4 (severe romantic lonely). Welch’s statistic was also significant for satisfaction with life with (3, 307) = 16.4, *p* < 0.001, and η^2^ = 0.05. The post hoc Games–Howell test indicated significant differences in life satisfaction scores across profiles (all *p*-values < 0.05) except for profile 2 (moderate romantic lonely) vs. profile 3 (moderate social lonely) and profile 3 vs. profile 4 (severe romantic lonely).

In Table 5, the multinomial logistic regression showed that being female decreases the likelihood of being classified as severe romantic lonely (ORs: 0.49, *p* < 0.001). Social isolation increased the likelihood of being classified as social and emotional lonely (ORs: 2.12, *p* < 0.05). Being in a relationship increased the likelihood of being classified as moderate social lonely (ORs: 9.22, *p* < 0.001) and decreased the likelihood of being classified as severe romantic lonely (ORs: 0.42, *p* < 0.001) as opposed to moderate social lonely. In terms of education stream, as opposed to moderate romantic lonely, students from the arts (ORs: 0.48, *p* < 0.001), commerce (ORs: 0.43, *p* < 0.05), and law (ORs: 0.42, *p* < 0.05) have a lower likelihood of being characterized as social and emotional lonely. Being a PhD student increases the likelihood of membership in the moderate social lonely group (ORs: 4.05, *p* < 0.05). Among the Big Five variables, agreeableness decreases the likelihood of being characterized as social and emotional lonely as opposed to moderate romantic lonely (ORs: 0.78, *p* < 0.001). As opposed to moderate romantic lonely, neuroticism increases the likelihood of membership in the social and emotional lonely (ORs: 1.40, *p* < 0.001), moderate social lonely (ORs: 1.24, *p* < 0.01), and severe romantic lonely groups (ORs: 1.23, *p* < 0.001).

## 4. Discussion

The study aimed to understand multi-dimensional loneliness among a representative sample of university students. The study’s objectives were to (a) use a person-centric approach to identify various profiles of lonely university students; (b) to see the difference between the identified profiles across various mental health and well-being indicators; and (c) to see whether demographic variables and Big Five personality variables can be used to predict profile membership. The findings show that a four-profile solution describes the sample adequately. The first profile comprised about one-third of the participants and scored the highest in family loneliness, moderate romantic loneliness, and had the highest social loneliness score. The profile was named ‘social and emotional lonely’ (SEL). The second profile scored the lowest on family loneliness and social loneliness, and moderately on romantic loneliness. Based on the scores on romantic loneliness, the group was given the designation ‘moderate romantic lonely’ (MRL). The third profile was the smallest homogenous group and scored low on family loneliness, the lowest on romantic loneliness, and moderately on social loneliness. This profile was denoted as ‘moderate social lonely’ (MSL). The fourth profile comprised more than one-third of the participants and scored low on family loneliness, moderately on social loneliness, and had the highest score for romantic loneliness. Compared to the other three profiles, the defining feature of this group were the highest levels of romantic loneliness. Therefore, the group was denoted as ‘severe romantic lonely’ (SRL).

The results show the existence of a latent structure within loneliness. The four-profile model is consistent with other studies which revealed three to five profiles of loneliness among young adults [25,27,29,33]. The four profiles extracted in the present study can also result within the context of culture. Past studies have shown that cultural differences influence the number of profiles even if the same standardized scales are used. For example, Shevlin and colleagues identified four lonely profiles in Northern Ireland, whereas a study by Zhang and colleagues in China reported three distinct profiles [26,32]. The profiles were also distinctive in the varying sources of loneliness: family, romantic, and social. These findings support the claim made by multi-dimensional theorists who have argued against classifying individuals solely as lonely or non-lonely based on arbitrary cut-off scores without considering the various sources of loneliness [16,29,58,59].

The current study’s findings also show that a specific profile of university students reported emotional (family and romantic) and social loneliness ranging from moderate to high intensity. The findings are consistent with Hyland et al. [29] who reported that 1/8 of U.S. adults feel emotional and social loneliness. They also found that a multi-dimensional approach can identify more than twice the number of at-risk individuals when compared to a unidimensional approach. An interesting finding of the study is that none of the profiles show low levels of loneliness across all dimensions. Past latent profile studies on loneliness have identified at least one profile with low scores on loneliness. For example, a study in New Zealand reported a low-loneliness group, the largest among the four identified profiles [31]. One potential explanation could be the relational closeness in collectivistic contexts such as India. As noted by Lykes and Kemmelmeier [60], individuals in collectivistic societies tend to derive their sense of identity and well-being from embeddedness within family and community relationships. Consequently, even modest disruptions in close relations such as separation from family during university years may lead to higher levels of loneliness.

The next objective of the study was to examine whether the identified profiles differ across multiple indicators. The study showed that all dimensions of loneliness are positively associated with depression. This aligns with previous studies on the relationship between loneliness and depression [61,62,63]. Few theorists believed loneliness and depression might overlap [64,65,66]. However, the current study shows a low-to-moderate relationship between loneliness and depression, suggesting that loneliness and depression are distinct. Findings show that an individual with moderate-to-high levels of loneliness across family, romantic, and social loneliness experiences the highest levels of depression. The findings align with past research on multi-dimensional loneliness [25,29]. This finding was consistent during COVID-19, when young adults who experienced high levels of loneliness also scored high on depression [27].

The findings of the current study reveal that profile 1 (social and emotional lonely) experiences the highest levels of anxiety, and profile 2 (moderate romantic lonely) experiences the lowest levels of anxiety. The findings do not show any profile experiencing different levels of depression and anxiety; the group that experienced the most depression also experienced the highest anxiety levels. The findings of the current study also reveal that profile 1 (social and emotional lonely) experiences the highest levels of stress, followed by profile 4 (severe romantic lonely), profile 3 (moderate social lonely), and profile 2 (moderate romantic lonely) experiences the lowest levels of stress. Past research has also shown that emotional loneliness (family and romantic) and social loneliness are associated with poorer psychological health [29]. Previous studies have also reported that lonely individuals report greater stress levels than non-lonely counterparts even if the stressors are similar in frequency and intensity [67,68,69].

Similarly, the profiles differentiated on the measure of life satisfaction. Moderate romantic lonely (MRL) scored the highest on life satisfaction, whereas the social emotional lonely (SEL) profile scored the lowest on life satisfaction. The findings align with other studies, which report significant differences in levels of life satisfaction among lonely profiles [31]. The moderate romantic lonely (MRL) profile scored the highest on social support levels, followed by severe romantic lonely (SRL) and social and emotional lonely (SEL). The findings align with previous studies in which individuals with high levels of social support are classified in low-scoring loneliness profiles [26]. Past research has also shown that support from family, friends, and teachers is a protective factor for university students [70]. One possible reason is that the lonely groups have lower social capital than non-lonely profiles [71]; therefore, they are stuck in a loop of poor social relationships.

The next objective of the study was to see whether variables such as gender, social isolation, relationship status, education characteristics, and the Big Five personality variables can predict profile membership supporting the discriminant validity of the loneliness profiles. The two loneliest groups—social and emotional lonely (SEL) and severe romantic lonely (SRL)—have a higher composition of males than females. Past studies have shown similar results where the loneliest groups show a higher composition of men [27,71]. Individuals from social and emotional lonely (SEL) were twice as likely to be socially isolated. One possible explanation for the university students showing high membership in the SEL profile is that socially isolated individuals who are not part of a strong social network experience more loneliness [72]. Being in a relationship emerged as a strong protective factor against severe romantic loneliness. The high probability of ‘committed’ students being classified in the moderate social lonely group, which scored low on family loneliness, the lowest in romantic loneliness, and moderately in social loneliness, is in line with past findings which reported that being single increases the chances of loneliness [9] and a committed relationship protects against loneliness [8].

Regarding the study year, being a Ph.D. student decreases the probability of being classified in social emotional lonely profile. This is in line with past studies which have shown that year of education significantly predicts loneliness [9,26]. Regarding the profile membership of stream of education, students pursuing arts, commerce, and law degrees were less likely to be classified in the in social emotional lonely profile. The findings are contrary to previous findings, which reported that undergraduates from social science (humanities) in Germany scored higher on social loneliness than students from other disciplines [8].

Among the Big Five personality traits, extraversion was not a significant predictor of profile membership. Previous latent profile studies have reported mixed findings. For example, a latent profile study reported that extraversion is significantly related to low-loneliness profiles [31]. However, another study during COVID-19 in China found that extraversion was not a significant predictor of profile membership [26]. Neuroticism was positively related to all dimensions of loneliness, which aligns with past studies that show a positive relationship between neuroticism and loneliness [73,74,75,76]. Neuroticism was also the strongest significant predictor of profile membership. The findings align with other latent profile studies that reported the highest level of neuroticism among high-scoring lonely profiles [26,31,77]. A possible explanation of the significant prediction of neuroticism on profile membership is the gender composition in social and emotional profile, which has a higher composition of male university students. For example, a recent meta-analysis has shown that young male adults score higher in neuroticism than their female counterparts [78]. Agreeableness was also a significant predictor of profile membership. Social emotional lonely (SEL) students are less agreeable. Past research has shown that loneliness is a personality-like trait. However, few studies have conclusive evidence to support the claim. The prediction of profile membership can be explained by Peplau and Perlman [64] with three possible indirect explanations for how personality may affect the experience of loneliness. First, certain personality traits score high in negative social attractiveness, for example, neuroticism, which was the strongest predictor of profile membership. Second, personality influences interactional behaviour. Lonely individuals act in a way that keeps them stuck in a loop of loneliness. Third, personality traits affect reactions to changes in social relations. These three hypotheses suggest that personality influences the formation and maintenance of satisfying relationships, affecting loneliness.

## 5. Implications, Limitations, and Conclusions

### 5.1. Implications

The research explored loneliness from a less-researched perspective through latent profile analysis and showed that different profiles of lonely individuals vary across mental health indicators. Those individuals who experience high levels of loneliness across multiple dimensions are most depressed, anxious, and stressed. They also had low social support and lower life satisfaction than other profiles. The study highlights the importance of considering the social and emotional aspects of loneliness among university students. Focusing on the unique experiences of the social and emotional lonely profile challenges traditional definitions of loneliness that often focus solely on the absence of social connections. This expanded conceptualization offers a more nuanced understanding of loneliness and emphasizes the role of emotional connection in the university context.

The practical implication of the study is identification of characteristically different profiles of lonely individuals and the emphasis on targeted support programmes among university students. With accurate diagnosis, student welfare departments can run special initiatives to help students connect; for example, students showing high social loneliness may benefit from structured peer network initiatives, interest-based clubs, and mentoring programmes that help expand their social circles. Students with high romantic loneliness may require counselling services and relationship skills workshops to enhance intimacy and emotional communication. Students experiencing family loneliness may respond better to family-oriented counselling programmes aimed at strengthening emotional resilience and alternative support networks. Implementing these practical strategies can improve student well-being, foster a sense of belonging, and create supportive campus environments that mitigate feelings of loneliness.

### 5.2. Limitations and Future Directions

There were a few limitations in the research. First, the participants were selected using the convenience sampling method from Uttar Pradesh, India; therefore, the generalizability to other students or populations is limited. Second, the study assessed three sources of loneliness (family, romantic, and social). However, including more sources of loneliness, such as transient, chronic, and existential loneliness could have yielded more comprehensive profiles. Third, the current research did not account for the duration of feeling lonely and the frequency of lonely periods. Such data could have yielded different profiles. Fourth, due to the paucity of time, the researcher could not validate the number of profiles identified in the study by replicating the profiles on similar samples. Future studies are encouraged to replicate the findings of several profiles on a similar sample. Fifth, the profiles were made in a snapshot since this is a cross-sectional study. Longitudinal studies could have yielded different profiles based on the transient or severe loneliness of individuals. Sixth, different samples, including professional groups of medical students, could yield different profiles. Seventh, a notable limitation concerns the internal reliability of the BFI-10 which may have attenuated the strength of personality-based predictions.

There are several possible research avenues that researchers can explore. The current research supports the multi-dimensional nature of loneliness beyond emotional and social loneliness, such as evolutionary loneliness. Most of the time, these approaches have been used independently. Future studies can incorporate more types of loneliness, and this may yield novel findings. Therefore, it is essential to identify the strengths of theoretical approaches and integrate them to explain loneliness holistically. Future studies could focus on finding profiles in the context of family composition; for example, whether family loneliness profiles exist within a particular family composition. Identifying such profiles may provide information about loneliness across generations. Future research can attempt to design questionnaires measuring transient and chronic loneliness. Considering the transient nature of loneliness, future work on latent profile analysis should focus on loneliness ratings in frequency and intensity within a defined time slot. Moreover, examining responses over multiple categories should work to provide more precision in the reporting of loneliness. Future research can focus more on the experiential aspect of loneliness.

### 5.3. Conclusions

Using latent profile analysis, the study examined whether different profiles of lonely university students exist among a representative sample of university students. Moreover, it examined whether the profiles differed across indicators such as depression, anxiety, stress, social support and life satisfaction. Lastly, the aim was to assess whether profile membership and odds ratio can be predicted based on the demographic variables (gender, social isolation, relationship status, and education characteristics) and Big Five personality traits (extraversion, agreeableness, openness, conscientiousness, and neuroticism). The results of the latent profile analysis showed four latent profiles of varying loneliness. The profiles differed across various indicators. The profile scoring highest on depression, anxiety, and stress also scored the lowest on life satisfaction and social support. Regarding profile membership, females were less likely to be characterized as members of the severe romantic lonely profile. Being in a relationship decreased the likelihood of being in severe romantic lonely profile. Among the Big Five personality traits, neuroticism was the strongest significant predictor of profile membership followed by agreeableness. In conclusion, the findings of this study provide valuable insights into the various dimensions of loneliness experienced by university students and their association with demographic factors, mental health outcomes, and personality traits.

## Figures and Tables

**Table 1:** Correlations for study variables.

	Fam L	Rom L	Soc L	Support	Extra	Agree	Consc	Neuro	Open	Dep	Anx	Stres	Sat	Mean (S.D.)
Fam L	1													11.75 (5.88)
Rom L	0.08 *	1												20.99 (7.54)
Soc L	0.32 ***	0.12 ***	1											16.14 (6.76)
Support	−0.49 ***	−0.31 ***	−0.52 ***	1										62.77 (15.43)
Extra	−0.11 ***	−0.15 ***	−0.15 **	0.18 ***	1									6.42 (1.99)
Agree	−0.16 ***	0.03	−0.10 **	0.11 **	0.02	1								7.10 (1.70)
Consc	−0.13 ***	−0.07 *	−0.02	0.14 ***	0.21 ***	0.05	1							6.46 (1.85)
Neuro	0.23 ***	0.11 ***	0.18 ***	−0.20 ***	−0.32 ***	−0.08 *	−0.21 ***	1						5.89 (1.99)
Open	−0.13 ***	−0.01	−0.04	0.11 ***	0.03	0.10 **	0.23 ***	−0.09 **	1					6.79 (1.66)
Dep	0.31 ***	0.20 ***	0.24 ***	−0.23 ***	−0.20 ***	−0.02	−0.28 ***	0.38 ***	−0.09 **	1				13.43 (4.52)
Anx	0.24 ***	0.10 **	0.15 ***	−0.18 ***	−0.12 ***	−0.02	−0.22 ***	0.36 ***	−0.08 *	0.71 ***	1			13.51 (4.43)
Stres	0.21 ***	0.11 ***	0.16 ***	−0.15 ***	−0.11 ***	−0.02	−0.21 ***	0.38 ***	−0.03	0.74 ***	0.77 ***	1		14.95 (4.11)
Sat	−0.26 ***	−0.26 ***	−0.18 ***	0.41 ***	0.20 ***	0.03	0.23 ***	−0.27 ***	0.08 *	−0.37 ***	−0.26 ***	−0.30 ***	1	21.50 (7.32)

Note: * *p* < 0.05, ** *p* < 0.01, *** *p* < 0.001; Fam L = family loneliness, Rom L = romantic loneliness, Soc L = social loneliness, Support = social support, Extra = extraversion, agree = agreeableness, consc = consciousness, neuro = neuroticism, open = openness, Dep = depression, Anx = anxiety, Stres = stress, Sat = satisfaction with life.

**Table 2:** Profile comparison and summary of models fit of the latent profile analysis.

Model	Profiles	BIC	AIC	SABIC	Bootstrap LRT_*p*	Entropy	Prob (Min–Max)
1	1	18,206	18,177	18,187	-	1.00	-
1	2	18,086	18,038	18,055	0.009	0.52	0.83–0.87
1	3	18,059	17,992	18,015	0.009	0.59	0.78–0.85
1	4	17,987	17,901	17,930	0.009	0.60	0.72–0.85
1	5	18,015	17,909	17,945	0.891	0.51	0.23–0.85
1	6	18,022	17,896	17,939	0.019	0.56	0.30–0.86
2	1	18,206	18,177	18,187	-	1.00	-
2	2	17,712	17,649	17,670	0.009	0.89	0.97–0.97
2	3	17,671	17,574	17,607	0.009	0.88	0.83–0.97
**2**	**4**	**17** **,624**	**17** **,494**	**17** **,538**	**0.009**	**0.73**	**0.78–0.98**
3	1	18,112	18,069	18,084	-	1.000	-
3	2	18,096	18,033	18,055	0.009	0.593	0.86–0.89
3	3	18,079	17,997	18,025	0.009	0.585	0.78–0.85
3	4	17,992	17,891	17,926	0.009	0.623	0.71–0.85
3	5	18,020	17,900	17,940	0.970	0.525	0.20–0.85
3	6	18,017	17,877	17,925	0.009	0.602	0.29–0.85
6	1	18,112	18,069	18,084	-	1.000	-
6	2	17,732	17,641	17,672	0.009	0.877	0.87–0.96
6	3	NaN	NaN	NaN	NaN	NaN	NaN
6	4	17,679	17,491	17,555	NaN	0.741	0.75–0.98

Note: Fit indices across different profiles with the best model highlighted in bold.

**Table 3:** Characteristics and nomenclature of the four profiles.

Profiles	Participants *n* (%)	Characteristics	Nomenclature
Profile 1	286 (31.4%)	Highest family loneliness Moderate romantic loneliness Highest social loneliness	Social and emotional lonely (SEL)
Profile 2	217 (23.8%)	Lowest family loneliness Moderate romantic loneliness Lowest social loneliness	Moderate romantic lonely (MRL)
Profile 3	75 (8.2%)	Low family loneliness Lowest romantic loneliness Moderate social loneliness	Moderate social lonely (MSL)
Profile 4	334 (36.6%)	Low family loneliness Highest romantic loneliness Moderate social loneliness	Severe romantic lonely (SRL)

**Table 4:** Differences between profiles and mental health indicators.

Variables	Social and Emotional Lonely (SEL) (*n* = 286)	Moderate Romantic Lonely (MRL) (*n* = 217)	Moderate Social Lonely (MSL) (*n* = 75)	Severe Romantic Lonely (SRL) (*n* = 334)	F	η^2^	Post Hoc (Games-Howell)
	M (S.D.)	M (S.D.)	M (S.D.)	M (S.D.)			
Depression	15.32 (4.88)	11.47 (3.81)	11.96 (3.73)	13.40 (4.12)	35.74 ***	0.11	1 > 2 ***, 1 > 3 ***, 1 > 4 ***; 4 > 2 **, 4 > 3 *
Anxiety	14.91	12.21	12.75	13.32	17.1 ***	0.05	1 > 2 ***, 1 > 3 **, 1 > 4 ***; 4 > 2 **, ns elsewhere
Stress	16.15	13.61	14.63	14.88	15.4 ***	0.05	1 > 2 ***, 1 > 3 *, 1 > 4 ***; 4 > 2 **, ns elsewhere
Social Support	53.38	72.77	70.53	62.56	98 ***	0.24	2 > 1 ***, 3 > 1 ***, 4 > 1 **; 2 > 4 ***, 3 > 4 ***; 2 = 3 (ns)
Life Satisfaction	19.44	23.75	23.40	21.38	16.4 ***	0.05	2 > 1 ***, 3 > 1 ***, 4 > 1 **; 2 > 4 ***; 3 = 4 (ns); 2 = 3 (ns)

Note: * *p* < 0.05, ** *p* < 0.01, *** *p* < 0.00, ns = not significant; M = arithmetic mean; SD = standard deviation, F = Welch’s F, η^2^ = eta squared.

**Table 5:** Posterior profile membership of demographic variables and Big Five personality traits.

	Social and Emotional Lonely vs. Moderate Romantic Lonely			Moderate Social Lonely vs. Moderate Romantic Lonely			Severe Romantic Lonely vs. Moderate Romantic Lonely		
Variables	Estimates	*p*	Odds Ratio (95% CI)	Estimates	*p*	Odds Ratio (95% CI)	Estimates	*P*	Odds Ratio (95% CI)
Female	−0.32	0.124	0.73 (0.49–1.09)	−0.09	0.760	0.91 (0.50–1.67)	−0.71	<0.001	0.49 (0.34–0.72)
Socially Isolated	0.75	0.005	2.12 (1.25–3.61)	−0.27	0.563	0.76 (0.31–1.90)	0.16	0.548	1.17 (0.70–1.97)
In a relationship	0.10	0.687	1.10 (0.68–1.78)	2.22	<0.001	9.22 (4.99–17.04)	−0.87	<0.001	0.42 (0.25–0.70)
Arts	−0.74	0.001	0.48 (0.31–0.74)	−0.61	0.075	0.55 (0.28–1.06)	−0.06	0.781	0.94 (0.62–1.44)
Commerce	−0.85	0.012	0.43 (0.22–0.83)	−0.81	0.124	0.45 (0.16–1.25)	−0.47	0.148	0.63 (0.33–1.18)
Law	−0.87	0.033	0.42 (0.19–0.93)	−0.61	0.302	0.54 (0.17–1.73)	−0.04	0.917	0.96 (0.46–2.00)
Masters	−0.31	0.137	0.73 (0.49–1.10)	−0.00	0.998	1.00 (0.54–1.84)	0.04	0.845	1.04 (0.71–1.51)
PhD	0.13	0.835	1.14 (0.34–3.75)	1.40	0.040	4.05 (1.06–15.41)	0.61	0.271	1.83 (0.62–5.38)
Extraversion	0.00	0.962	1.00 (0.90–1.11)	0.03	0.655	1.04 (0.89–1.21)	−0.06	0.207	0.94 (0.86–1.03)
Agreeableness	−0.24	<0.001	0.78 (0.70–0.88)	−0.13	0.153	0.88 (0.74–1.05)	−0.10	0.087	0.91 (0.81–1.01)
Consciousness	−0.10	0.088	0.91 (0.81–1.01)	−0.05	0.571	0.95 (0.81–1.12)	0.02	0.702	1.02 (0.92–1.13)
Neuroticism	0.34	<0.001	1.40 (1.26–1.56)	0.21	0.007	1.24 (1.06–1.45)	0.20	<0.001	1.23 (1.11–1.35)
Openness	−0.11	0.067	0.89 (0.79–1.01)	0.10	0.260	1.11 (0.93–1.32)	−0.01	0.863	0.99 (0.99–0.89)

Note: Reference groups: moderate romantic lonely (profile 2), male, not socially isolated, single, graduate student.

## Data Availability

Data will be made available upon request.
